# Impact of the compact county medical service community policy on healthcare resource allocation and healthcare service utilization in village clinics: a difference-in-differences analysis in rural Sichuan, China

**DOI:** 10.3389/fpubh.2026.1826414

**Published:** 2026-06-01

**Authors:** Meng Li, Liping Fu, Anhui Mo, Longhui Li, Yujie Lin

**Affiliations:** 1 College of Management and Economics, Tianjin University, Tianjin, China; 2 Ma Yinchu School of Economics, Tianjin University, Tianjin, China

**Keywords:** compact county medical service community policy, difference-in-differences analysis, healthcare resource allocation, healthcare service utilization, rural Sichuan, China, village clinics

## Abstract

**Introduction:**

This study explores the impact of the compact county medical service community policy on healthcare resource allocation and healthcare service utilization in rural Sichuan, China. The policy was implemented in stages in 2017, 2018, 2019, 2020 and 2023.

**Method:**

Data were sourced from the Sichuan Health Statistics Yearbooks and Sichuan Statistical Yearbooks (2014–2023). We employed a staggered difference-in-differences propensity score matching analysis with two-way fixed effects, using data from 1,800 county-year observations in rural Sichuan over the period 2014–2023. After eliminating missing values, the treatment and control groups comprised 36 and 144 counties, respectively.

**Results:**

The implementation of compact county medical service community policy was associated with the successful optimization of healthcare resource allocation in village clinics, predominantly attributed to statistically significant increases in the number of healthcare workers and village clinics by an average of 0.163 (8.3%) and 0.089 (6.2%) per 1,000 rural population, respectively. Additionally, healthcare service utilization in village clinics substantially improved. Detailed analysis revealed a statistically significant average increase of 0.164 (6.8%) per 1,000 rural population in the number of healthcare consultations. The number of emergency consultations also increased by an average of 0.159 (6.9%) per 1,000 rural population.

**Discussion:**

Our findings highlight the beneficial impacts of implementing compact county medical service community policy on healthcare resource allocation and healthcare service utilization in rural areas, particularly in terms of village clinics. The findings have critical policy implications for enhancing healthcare resource allocation and healthcare service utilization in rural China.

## 1 Introduction

Recently, the healthcare system in China has undergone significant systemic health reforms to provide equitable and efficient healthcare services to citizens nationwide (1). A vital component of the reforms was restructuring the healthcare system in primary-level areas, an initiative launched in 2009 (2). This reform focused on establishing primary healthcare service institutions (e.g., township health centers and village clinics) and improving the competence of healthcare workers. These measures aimed to enhance the healthcare service capability in primary-level areas and encourage patients to seek local healthcare services (3, 4). However, the quality of healthcare services in China has remained suboptimal, especially in rural areas (5), and trust in healthcare services provided by primary care institutions in rural areas remains low (6). Most rural patients tend to bypass local primary healthcare and seek care at higher level hospitals in nearby urban regions (7). To address this problem, the State Council and National Health Commission (8) launched the compact county medical service community policy (hereafter ‘CCMSC policy’) in 2019. It is China’s largest public health service reform policy, aimed at enhancing the healthcare service capability of primary care institutions, particularly in rural areas (8, 9).

The CCMSC policy aims to facilitate tiered allocation of healthcare resources (e.g., doctors, healthcare beds and expenditure) from county-level cities to rural areas via a three-level model of coordinative governance consisting of county-level public hospitals, township health centers and village clinics (9, 10). The policy has been implemented nationwide in stages to enable flexible collaborative governance, with policy pilots for each province and municipality in China (9). Pilot-testing the policy at a limited scale is helpful for identifying and resolving potential risks and problems. The Chinese government has divided the healthcare service system in primary-level areas into urban and rural components, which are organized differently (11). Urban primary care institutions comprise community healthcare centers and stations (12, 13). Rural primary care institutions include township health centers and village clinics (14). Village clinics play a crucial role in healthcare service delivery in rural areas and are the first-line healthcare service institutions offering basic and preventive healthcare to rural residents (15, 16).

The evolution of county-level healthcare resource integration has typically transitioned from loose medical alliances to compact medical communities, representing a fundamental shift in organizational architecture and management authority (5, 17). The loose medical consortium is primarily characterized as a horizontal collaborative mechanism based on technical support or referral protocols. In this model, county-level public hospitals, township health centers, and village clinics maintain independent administrative affiliations, financial accounting, and human resource management systems (18, 19). In the loose model, healthcare resource allocation is often passive and sporadic (18, 20). Owing to the lack of sustained incentives, support from higher-tier hospitals tends to be symbolic (e.g., occasional free clinics and lectures), making those healthcare resources difficult to effectively channel to the village level.

In contrast, the CCMSC policy facilitates a substantive vertical integration. Earlier studies (9, 17) indicate that the core of tight integration lies in breaking down administrative barriers to achieve unified management and allocation of key factors, including human resources, finances, and facilities. A coordinated governance mechanism plays an important role in the implementation of the CCMSC policy. Specifically, the county-level public hospitals as the lead unite township health centers and village clinics as a community of shared interests (18, 21). The county-level public hospitals as the lead are responsible not only for purchasing healthcare facilities, recruiting healthcare workers, and securing funding, but also for channeling the healthcare resources to rural healthcare service institutions (township health centers and village clinics) on demand. Moreover, the county-level public hospitals assume substantial operational management authority over healthcare resources allocated within the community (10). After implementing the CCMSC policy, township health centers and village clinics are no longer required to independently purchase costly medical equipment and recruit healthcare workers. Healthcare resources can flow dynamically among county-level public hospitals, township health centers, and village clinics (9). Ultimately, the management authority over remaining resources, such as financial budgets and the rotation of doctors to village clinics, is centralized at the county-level public hospitals for unified administration (21).

Existing studies of healthcare service system reforms in China have undertaken limited exploration of the CCMSC policy in primary-level areas, primarily examining its efficiency (e.g., resource allocation and inpatient expenditure per capita), utilization (e.g., the number of healthcare consultations and surgery rate), and the challenges caused by rural healthcare system reforms (9, 10, 22). Few studies have evaluated the general impact of the CCMSC policy. Wu et al. (21) used a case study to evaluate the reform in Zhejiang province, China. Ding and Zhou (9) empirically analyzed the impact of the compact county medical communities on hierarchical diagnosis and treatment and indexes of healthcare service utilization in the entire primary-level area in Sichuan. Studies have highlighted that establishing more flexible mechanisms of healthcare service deliveries is essential to optimize the healthcare resource allocation in primary-level areas and enhance the accessibility of healthcare services for local residents (21), particularly for rural residents. The introduction of the CCMSC policy represents a major healthcare policy innovation in this regard. However, empirical research on the impacts of the CCMSC policy remains limited, particularly in terms of rural areas.

Additionally, existing literature on healthcare services provided by village clinics is limited, with studies largely focusing on healthcare resource allocation, healthcare-seeking behavior of rural residents, and specific disease management. Early studies (23, 24) performed descriptive analysis and ANOVA to separately analyze the general effect of the new healthcare system reform on job satisfaction and turnover intention of doctors in village clinics, finding a decrease in job satisfaction and an increase in turnover intention, indicating that the policy had a negative effect on the allocation of doctors in village clinics. Chen et al. (16) explored the changes in utilization of village clinics in China’s rural areas through detailed Kitagawa-Oaxaca-Blinder decompositions. They primarily found a decrease in the number of treated patients in village clinics, with an increasing number of individuals opting for self-treatment over clinical care. Chu et al. (25) assessed the general impact of a telemedicine-based, village doctor-led integrated healthcare model on atrial fibrillation care, finding improved treatment adherence and enhanced clinical outcomes compared to usual care. However, research is scarce on healthcare resource allocation and healthcare service utilization in village clinics. Although Ding and Zhou (9) empirically analyzed the impact of county medical community policy on hierarchical diagnosis and treatment in county-level areas, their study was limited to observations in entire primary-level areas. A noticeable research gap still exists in understanding the impact of the CCMSC policy on healthcare resource allocation and healthcare service utilization in rural areas, specifically in terms of village clinics. To our knowledge, our paper is the first to explore this aspect.

In this study, we employed a staggered difference-in-differences (DID) approach to evaluate the impact of the CCMSC policy on healthcare resource allocation and healthcare service utilization in village clinics of rural Sichuan, using data from Sichuan Health Statistics Yearbooks and Sichuan Statistical Yearbooks for the period 2014–2023. This study aims to contribute to advancing understanding of the implications of the CCMSC policy for rural healthcare system reforms in Sichuan, as well as in the rest of the nation, to offer valuable insights for future healthcare reforms in China.

## 2 Methods

### 2.1 Policy background

In 2015, the General Office of the State Council in China issued the policy “Guiding Opinions on Promoting the Construction of a Hierarchical Diagnosis and Treatment System.” This policy aims to explore medical consortium formation and develop a regional collaborative governance model with clearly defined roles, rights, and benefits (1). In 2017, “Guiding Opinions on the Construction and Development of Medical Consortia” was released, which classified medical consortia into four categories: city medical groups, inter-regional specialty alliances, county medical consortia, and telemedicine collaboration networks (9). The focus was on exploring the county medical consortia to optimize the allocation of healthcare resources and enhance the healthcare service capability in primary-level areas to construct the hierarchical diagnosis and treatment system.

**Figure 1:**
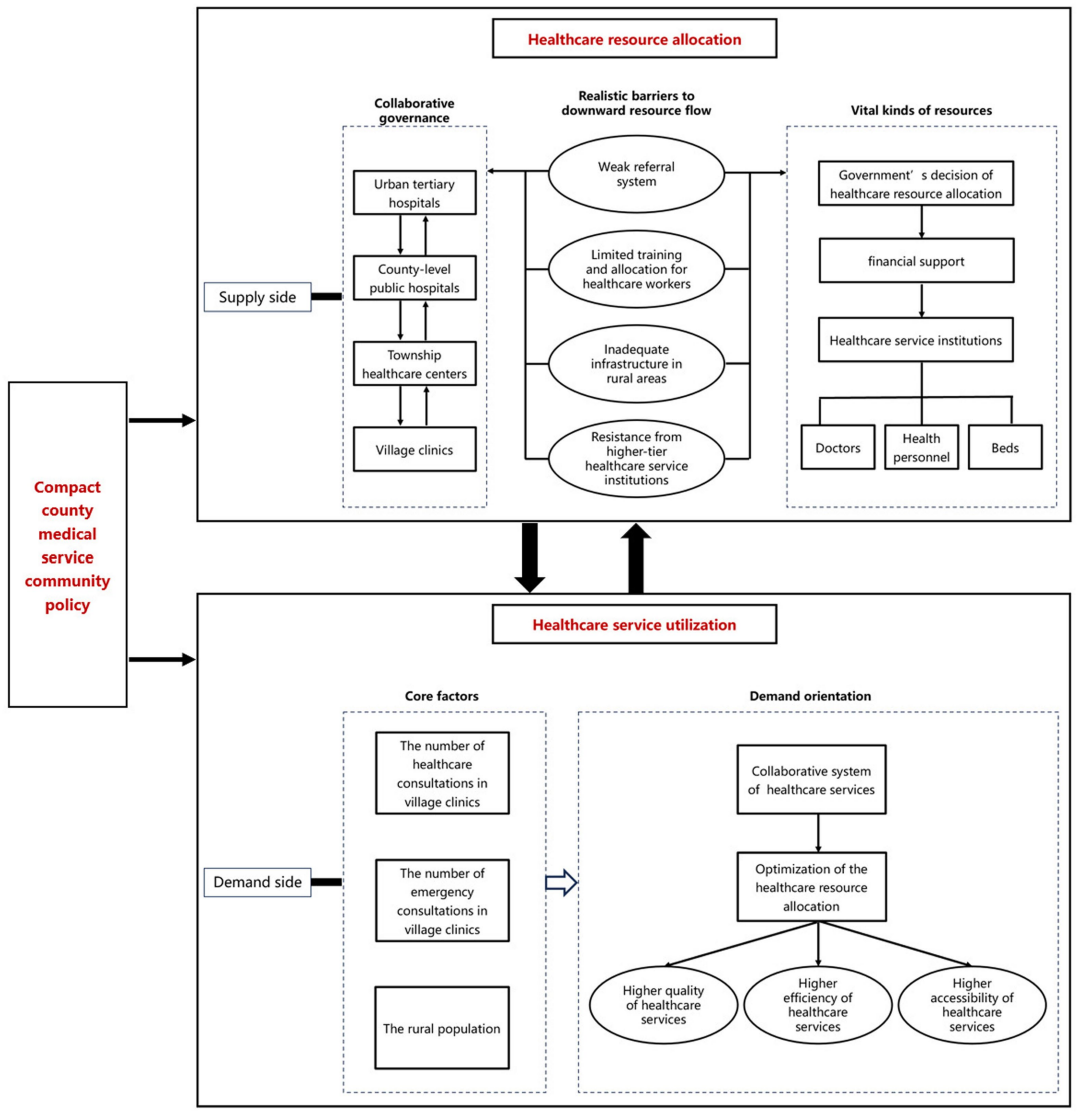
Conceptual framework for the impact of the compact county medical service community policy on healthcare resource allocation and healthcare service utilization.

In 2019, the General Office of the State Council and the National Health Commission jointly released the policy document “Notice on Promoting the Construction of Compact County Medical Service Community.” The CCMSC policy aims to establish an integrated management model at the county and township levels to achieve a tiered allocation of healthcare resources (e.g., doctors, health personnel and expenditure) from county-level cities to rural areas via a three-level model of coordinative governance, and promote the realization of a hierarchical diagnosis and treatment system (9, 10). Figure 1 shows the conceptual framework of the CCMSC policy. The policy operates through a collaborative governance system of healthcare resource allocation from urban tertiary hospitals to village clinics in rural areas. As shown in Figure 1, government distributes capital to urban tertiary hospitals and county-level public hospitals. Using this financial support, the county-level public hospitals recruit healthcare workers (e.g., doctors and health personnel) and purchase healthcare beds based on the demand of health care among the local population. Next, these health workers, beds, and the remaining capital are systematically allocated to township healthcare centers and village clinics. Specifically, the county-level public hospitals act as the lead, the township health centers are the central hubs, and village clinics form the foundation. The collaborative governance model can optimize the allocation of healthcare resources in primary-level areas, particularly in rural areas, and promote balanced allocation of healthcare resources between regions. Moreover, local patients can also obtain higher-quality, efficient, and accessible healthcare services.

However, as shown in Figure 1, there are some realistic barriers to the implementation of the CCMSC policy, including a weak referral system, limited training and allocation of healthcare workers in rural areas, inadequate infrastructure in rural areas, and resistance from higher-tier healthcare service institutions.

Additionally, in September 2019, the Sichuan Health Commission issued the Implementation Plan for the Construction of Compact County Medical Service Community in Sichuan Province. The official document identifies 37 counties designated as piloted counties for implementing the policy (accounting for 20% of all counties in Sichuan). Each city has one or more piloted counties. However, the CCMSC policy was not implemented concurrently nationwide, but rather in stages in 2017, 2018, 2019, 2020, and 2023. In Sichuan Province, 1 pilot county implemented the CCMSC policy beginning in 2017, 3 counties began in 2018, 19 began in 2019, 13 began in 2020, and 1 began in 2023. Supplementary Table S1 presents a list of the 37 pilot counties and when they implemented the CCMSC policy.

### 2.2 Sample and data

Data were collected from the Sichuan Health Statistics Yearbooks and Sichuan Statistical Yearbooks between 2014 and 2023. The Sichuan Health Statistics Yearbooks were officially launched by the Sichuan Health Commission since 2012. They contain provincially representative healthcare data collected annually with delayed release to comprehensively monitor the development of the healthcare service system in Sichuan Province. The yearbooks compile different types of data on healthcare resources and services in all parts of Sichuan Province, especially in village clinics. The Sichuan Health Statistics Yearbooks use a four-sort healthcare service index for village clinics, with the first sort selecting the number of healthcare workers, the second selecting the number of village clinics, the third selecting the number of healthcare consultations, and the fourth sort selecting the number of emergency consultations. We obtained a wide range of healthcare-related data from the Sichuan Health Statistics Yearbooks, especially in primary-level areas, including the number of doctors, healthcare consultations, healthcare institutions, and healthcare beds. Village clinics are important healthcare institutions in primary-level areas. Hence, the integral development of healthcare services in primary-level areas directly affects the capability of village clinics. Additionally, the Sichuan Statistical Yearbooks have been officially compiled by the Sichuan government since 2006. They contain provincially representative economic and social development data, collected via similar sampling strategies and methods used for the Sichuan Health Statistics Yearbooks, with data collected for 22 fields, such as population, finance, and economy (26). We used the rural population and the per capita gross regional product of all places from the Sichuan Statistical Yearbooks. Many studies have used data from these two yearbooks to assess trends in the economy, population, and healthcare (9, 27, 28).

The data from the Sichuan Health Statistics Yearbooks for village clinics cover all 183 counties in Sichuan. Based on a document available on the official website of the State Council for the People’s Republic of China, 37 pilot counties (accounting for 20% of all counties in Sichuan) implemented the CCMSC policy (8); the remaining 144 were non-pilot counties and were assigned to the control group. To determine the selection criteria for the 37 pilot counties, we utilized a logit model to predict the probability of a county being selected as a pilot county using only pre-policy data (2014–2018). Subsequently, we merged these propensity score estimates with the full sample (2014–2023) to perform a weighted PSM-DID analysis, thereby mitigating potential selection biases in the treatment group. Results (Supplementary Table S2) indicated that the 37 pilot counties were selected based on their actual demand for healthcare services, instead of only economic development level, rural population, or current healthcare resources. We acknowledge that the pilot counties were not randomly assigned and likely selected based on the higher demand for pre-policy healthcare services, which introduces positive selection bias. To rigorously address this potential selection bias and endogeneity, we employed propensity score matching (PSM), using nearest-neighbor matching with a caliper, combined with difference-in-differences analysis (PSM-DID) (29). The matching significantly improved the covariate balance, reducing the standardized biases to below 10% for all variables (Table 1).

**Table 1:** Results from the PSM between treatment and control groups.

Variables	Unmatched (U)	Mean		%Bias	% reduct |bias|	t-test	
	Matched (M)	Treatment	Control			t	*p* value
Per capita gross regional product	U	1.31	1.12	34.9	71.4	4.09	0.000
	M	1.31	1.25	10.0		0.94	0.348
Doctors in primary-level areas	U	3.04	3.18	−19.6	96.3	−2.17	0.030
	M	3.03	3.04	−0.7		−0.08	0.937
Healthcare consultations in primary-level areas	U	3.24	2.82	37.1	85.4	4.51	0.000
	M	3.30	3.37	−5.4		−0.52	0.602
Healthcare service institutions in primary-level areas	U	1.01	1.20	−33.8	96.1	−3.60	0.000
	M	1.02	1.02	−1.3		−0.17	0.864
Healthcare beds in primary-level areas	U	1.62	1.58	7.0	37.0	0.85	0.396
	M	1.60	1.63	−4.4		−0.42	0.675
Rural population	U	234.66	227.95	3.8	−4.0	0.46	0.649
	M	234.65	241.63	−4.0		0.40	0.691

Sample	Pseudo *R^2^*	Likelihood ratio *chi^2^*	*p* value
Unmatched	0.035	31.70	<0.001
Matched	0.004	1.94	0.925

After eliminating the post-policy period (2019–2023), we conducted the PSM using nearest neighbor matching with a caliper using data during the pre-policy period (2014–2018). Counties in the treatment group were matched with those in the control group through propensity score, utilizing nearest neighbor matching with a caliper. In total, there were 873 observations, 178 observations in the treatment group and 695 in the control group. All covariates were measured before matching; % bias represents the mean standardized bias (74). In addition, after estimating the propensity scores, we set the caliper to 0.25 times the standard deviation of the propensity score (75). The standard deviation of the propensity score was 0.074. Hence, the caliper was 0.02.

We adopted a staggered DID approach to evaluate the causal impact of the CCMSC policy on healthcare resource allocation and healthcare service utilization in village clinics. This approach requires satisfying the parallel trends assumption (30). We argue that the selection bias primarily manifests as differences in initial levels rather than in growth trends. After matching counties with similar baseline characteristics via PSM, we ensured that the treatment and control groups were comparable at onset. Conditional on these covariates, it is reasonable to assume that the growth trajectories of both groups would have remained parallel in the absence of the CCMSC policy. Hence, for each indicator of healthcare resource allocation and healthcare service utilization, we assumed that the trends in the 37 pilot counties and the 144 non-pilot counties would have evolved similarly without the policy intervention (31).

To enhance data reliability before the formal data analysis, we conducted data preprocessing based on the following steps. First, owing to the lack of data for critical variables (e.g., the number of doctors in primary-level areas and rural population) for some years, we excluded two counties (Wuhou County and Leibo County), accounting for approximately 1% of the total sample. Second, Junlian County started to implement the CCMSC policy in October 2023 (32, 33). However, the data for this study were sampled between 2014 and 2023. Hence, the data for Junlian County could not be used to evaluate the impact of the CCMSC policy; therefore, we eliminated this pilot county. Third, in 2016, the Sichuan government adjusted the administrative boundary of Jianyang County from Ziyang City to Chengdu City. As Jianyang County was not a pilot location of the CCMSC policy, this adjustment did not affect the policy’s impact. Four, for ease of presentation, the raw frequencies of healthcare and emergency consultations in village clinics and healthcare consultations in primary-level areas were standardized to per 1,000 units. The final sample constituted a balanced panel dataset covering 36 treated and 144 control counties in rural Sichuan over the period 2014–2023. No county exited or entered during the data period. The treatment and control groups remained unchanged across all years.

### 2.3 Outcome variables

This study focuses on two primary outcomes: healthcare resource allocation and healthcare service utilization in village clinics. Healthcare resource allocation in village clinics was measured based on the number of village clinics and the number of healthcare workers—doctors and other healthcare personnel—employed by them, as suggested by previous research (16, 23). Healthcare service utilization in village clinics was measured by the number of healthcare and emergency consultations, as suggested by Ding and Zhou (9) and Wang et al. (34). Several studies show that healthcare resource allocation and healthcare service utilization are improving, and more patients are interested in local healthcare services (35–38). Conversely, decreased utilization might indicate lower-quality healthcare services, leading patients to seek services elsewhere. All outcome variables were divided by 1,000 rural population. Some key points concerning the two categories of dependent variables are detailed in Table 2.

**Table 2:** Definitions of the outcome variables.

Indicators	Variables	Definition
Healthcare resource allocation		
Y1	No. of healthcare workers in village clinics per 1,000 rural residents	No. of doctors, nurses and health personnel per 1,000 rural population of village clinics represents the main allocation of healthcare human resources in rural areas.
Y2	No. of village clinics per 1,000 rural residents	No. of village clinics per 1,000 rural population indicates the allocation of healthcare service institutions in rural areas.
Healthcare service utilization		
Y3	No. of healthcare consultations in village clinics per thousand rural residents	No. of healthcare consultations per 1,000 rural population represents frequency of local people coming to village clinics for healthcare service seeking.
Y4	No. of emergency consultations in village clinics per thousand rural residents	No. of emergency consultations per 1,000 rural population indicates the frequency of local people coming to village clinics for urgent healthcare service seeking at irregular time.

### 2.4 Covariates

We identified covariates associated with healthcare resource allocation and healthcare service utilization in village clinics in rural Sichuan based on the relevant literature (9, 39–41). These covariates were divided into three categories. First, per capita gross regional product can reflect the development quality of the economy in the pilot and non-pilot sites. We applied a logarithmic transformation to the variable, per capita gross regional product, in our model analysis. Second, rural population can reflect the demographic characteristics in rural areas. Third, Chen et al. (23) found that village clinics play an important role in providing healthcare services in primary-level areas. Other researchers have also demonstrated that in the primary healthcare service system in China, the village clinic is a vital part of rural healthcare service institutions (14). These covariates of the number of doctors, healthcare beds, and healthcare service institutions in primary-level areas can reflect the overall healthcare resource allocation in the studied areas. Furthermore, the number of healthcare consultations in primary-level areas can reflect the overall healthcare service utilization in those areas.

Additionally, we added two-way fixed effects of time and place for the year and pilot areas to examine the policy impact change in 2023 relative to the baseline year of 2019. Finally, we calculated the variance inflation factor (VIF) of all related independent variables and covariates. The VIF values of all variables were lower than 10, suggesting that there was no severe multicollinearity problem.

### 2.5 Statistical analysis

A difference-in-differences (DID) approach was employed to estimate the causal impact of the CCMSC policy on healthcare resource allocation and healthcare service utilization in village clinics in rural Sichuan, China (42). The DID approach is a natural experimental approach that allowed us to compare outcomes between treatment and control groups before and after implementing the CCMSC policy while controlling for potential confounding factors (43, 44). The causal impact of the intervention of the CCMSC policy is estimated by comparing the differences between two variations in outcomes, which are variations between the pre- and post-intervention periods within the treatment group and the pre- and post-intervention periods in the control group (45).

A staggered DID approach was used to estimate the causal impact of the CCMSC policy on healthcare resource allocation and healthcare service utilization in village clinics in the treatment group (46). Following Liu et al. (30) and Beck et al. (47), we conducted the regression described in Equation 1:

$$Y_{\mathrm{it}}=\alpha +\beta .\text{CCMS}C_{\mathrm{it}}+\sum_{h=1}^{\kappa }\gamma_{h}.X_{\mathrm{it}}^{h}+\lambda_{i}+\theta_{t}+\varepsilon_{\mathrm{it}}$$ (1)

where the outcome variable $Y_{\mathrm{it}}$ denotes a series of indicators of healthcare resource allocation and healthcare service utilization in village clinics, including the number of village clinics, healthcare workers, and healthcare and emergency consultations in village clinics in pilot counties i at year t . The intercept term, α , represents the baseline level of the outcome variable. For the key dependent variable, we established the grouping variable treatment and time dummy variable post . If a county belonged to the treatment group, treatment=1 ; otherwise, treatment=0 . If a county implemented the CCMSC policy in a given year, then post=1 ; otherwise post=0 . Then, we define CCMSC=treatment×post . Its coefficient β captures the treatment effect of the CCMSC policy. Additionally, a vector of covariates, X , is included in the model, such as per capita gross regional product and the number of doctors in the primary-level areas. $\gamma_{h}$ represents the coefficient of covariate $X^{h}$ ; h (h = 1, …, n) represents the serial number of covariates; and κ represents the total number of covariates. Two-way fixed effects of location and year are represented by $\lambda_{i}$ and $\theta_{t}$ , respectively. A random error term $\varepsilon_{\mathrm{it}}$ addresses unobserved factors, which might vary over time and affect the outcome.

Considering the disruptions caused by the COVID-19 pandemic (2020–2022), we conducted sensitivity analysis to further estimate whether the effect of CCMSC policy on healthcare resource allocation and healthcare service utilization in village clinics persisted independently of the pandemic lockdowns. This analysis is presented in the section that details robustness tests.

This study used publicly available data from the 2014–2023 Sichuan Health Statistics Yearbooks and Sichuan Statistical Yearbooks. None of the data involved personally identifiable information; therefore, ethical approval was not required.

## 3 Results

### 3.1 Descriptive statistics

Table 3 presents the mean values and standard deviations (SD) of all indicators of outcome variables and covariates for the treatment group (counties implementing the CCMSC policy) and the control group (counties not implementing the policy). The total sample size consisted of 1,800 county-year observations spanning 10 years. Following the removal of invalid samples, the treatment group included 36 counties (360 observations), while the control group comprised 141 counties (1,440 observations). Prior to policy implementation, the average numbers of village clinics and healthcare workers per 1,000 rural population in the treatment group were 1.36 and 1.92, respectively, compared to 1.54 and 1.98, respectively, in the control group. After the formal implementation of the policy in 2019, the yearly average number of healthcare workers in village clinics in the treatment group rose to 2.01 per 1,000 rural population, exceeding that of the control group (1.96). Meanwhile, the yearly average number of village clinics in the treatment group was 1.51 per 1,000 rural population, which was slightly lower than the control group but remained nearly identical to the control group’s pre-policy level (1.54). Furthermore, before formal policy implementation, the yearly average numbers of healthcare and emergency consultations in village clinics in the treatment group were 2.38 and 2.29 per 1,000 rural population, respectively, surpassing the control group’s figures of 1.86 and 1.75. Post-implementation, these averages increased to 2.43 and 2.34, remaining higher than those in the control group (1.75 and 1.66). Additionally, the mean rural populations in both the treatment and control groups decreased after formal policy implementation compared to the pre-implementation period. Nevertheless, the treatment group demonstrated higher yearly average healthcare service utilization, as measured by healthcare and emergency consultations in village clinics, relative to the control group. Table 3 indicates that while all outcomes showed significant improvement, the policy was implemented in stages across different pilot counties. Consequently, further testing via a formal staggered DID analysis is required to determine whether a causal relationship exists between the policy and the two primary outcomes.

**Table 3:** Descriptive statistics for the impact of the compact county medical service community policy in village clinics in each group of rural Sichuan (2014 to 2023).

Variables	Sample size: 1,800					
	Treatment group (*N* = 360)			Control group (*N* = 1,440)		
	2014–2018 (Obs = 180)	2019–2023 (Obs = 180)	Full period	2014–2018 (Obs = 720)	2019–2023 (Obs = 720)	Full period
Healthcare workers in village clinics	1.92 (0.67)	2.01 (0.78)	1.96 (0.73)	1.99 (0.89)	1.96 (0.99)	1.97 (0.94)
Village clinics	1.36 (0.56)	1.51 (0.69)	1.43 (0.63)	1.54 (0.83)	1.60 (0.95)	1.57 (0.89)
Healthcare consultations in village clinics	2.38 (1.30)	2.43 (1.39)	2.41 (1.35)	1.86 (1.50)	1.75 (1.47)	1.80 (1.49)
Emergency consultations in village clinics	2.29 (1.28)	2.34 (1.35)	2.32 (1.31)	1.75 (1.43)	1.66 (1.41)	1.71 (1.42)
Per capita gross regional product	1.31 (0.53)	1.72 (0.42)	1.52 (0.52)	1.12 (0.57)	1.59 (0.48)	1.36 (0.58)
Doctors in primary-level areas	3.04 (0.63)	3.56 (0.85)	3.30 (0.79)	3.18 (0.84)	3.72 (0.98)	3.45 (0.95)
Healthcare consultations in primary-level areas	3.34 (1.43)	3.68 (1.49)	3.51 (1.47)	2.82 (1.37)	3.02 (1.47)	2.92 (1.42)
Healthcare service institutions in primary-level areas	1.01 (0.40)	1.07 (0.49)	1.04 (0.45)	1.20 (0.65)	1.21 (0.69)	1.20 (0.67)
Healthcare beds in primary-level areas	1.62 (0.63)	1.94 (0.96)	1.78 (0.83)	1.58 (0.61)	1.80 (0.85)	1.69 (0.75)
Rural population/1,000	234.66 (175.29)	200.77 (150.13)	217.71 (163.85)	227.95 (177.03)	195.82 (149.54)	211.88 (164.59)

Y1 and Y2 separately represent the number of healthcare workers and village clinics in rural Sichuan. The numbers of village clinics, healthcare workers, healthcare and emergency consultations in village clinics are divided by 1,000 rural population. Per capita gross regional product, the numbers of doctors, healthcare institutions, beds and consultations in primary-level areas are divided by 1,000 total resident population. The raw numbers of healthcare and emergency consultations in village clinics and healthcare consultations in primary-level areas were converted to per 1,000 units.

Additionally, although the policy was implemented in stages for different pilot counties, the list of pilot counties was released formally in 2019. We observed that compared with the pre-policy period (2014–2018), there were decreases in the number of healthcare and emergency consultations per 1,000 rural population in the control group after formally implementing the policy in 2019. While the treatment group experienced an increase in healthcare service utilization in village clinics during the post-policy period (2019–2022), the downward trend in the control group may be attributed to the following broader national or regional factors that were not directly influenced by the CCMSC policy. First, economic fluctuations can significantly impact healthcare-seeking behavior for residents (48). A later study (49) emphasized that periods of economic downturn are often associated with reduced healthcare service utilization due to financial constraints among rural residents. Second, migration patterns could be an external factor, as rural-to-urban migration has been a persistent trend in China (50, 51). The population loss could potentially reduce the rural population and consultation rates in village clinics. Third, changes in healthcare-seeking behavior among rural residents might also contribute to the observed decline in healthcare service utilization in village clinics, such as seeking health care in higher-tier healthcare service institutions and increased reliance on self-care (16, 52). Finally, village clinics could be responsible for delivering primary healthcare for patients and facilitate referrals for some patients with serious diseases (5, 53). Owing to the COVID-19 pandemic, a portion of rural residents with conditions typically managed in village clinics had to transfer treatment to higher-tier healthcare service institutions, such as township health centers and county-level public hospitals, thereby leading to decreased utilization of village clinics.

### 3.2 Impact of the CCMSC policy on healthcare resource allocation in village clinics

According to the results presented in Table 4, first, the number of healthcare workers in village clinics experienced a statistically significant average increase of 0.118 (c. 6.0%) per 1,000 rural population in rural Sichuan after implementing the CCMSC policy. Second, the number of village clinics also exhibited a statistically significant average increase of 0.047 (c. 3.3%) per 1,000 rural population in rural Sichuan. These increases reflect a significant enhancement of healthcare resource allocation in village clinics in Sichuan. The enhancement can be primarily attributed to the growth of per capita gross regional product and an increase in the number of doctors and healthcare service institutions in primary-level areas. However, the number of healthcare consultations in primary-level areas and the rural population had a minor negative impact on the healthcare resource allocation in village clinics. The number of healthcare beds in primary-level areas also exhibited a statistically significant negative impact. This reflects macro-environmental changes in the reform of the current healthcare service system in China. With increasing numbers of healthcare consultations in primary-level areas, healthcare workers in village clinics were distributed to higher-level healthcare service institutions (township health centers and county-level public hospitals) to balance supply and demand in local healthcare (54). Moreover, village clinics act as outpatient hubs and primary triage centers in the primary healthcare service system (53). With the continuous outflow of rural residents from rural to urban areas, the negative effect of healthcare beds in primary-level areas indicated that inpatient demand is primarily concentrated in higher-level healthcare service institutions, such as township health centers and county-level public hospitals.

**Table 4:** Impact of compact county medical service community policy on healthcare resource allocation in village clinics.

Variables	Y1	Y2
DID	0.118^***^ (0.029)	0.047^***^ (0.016)
Per capita gross regional product	0.263^***^ (0.048)	0.137^***^ (0.027)
Doctors in primary-level areas	0.299^***^ (0.021)	−0.032^***^ (0.012)
Healthcare consultations in primary-level areas	−0.081^***^ (0.013)	−0.028^***^ (0.007)
Healthcare service institutions in primary-level areas	0.850^***^ (0.047)	1.380^***^ (0.027)
Healthcare beds in primary-level areas	−0.093^***^ (0.021)	−0.054^***^ (0.012)
Rural population	−0.001^***^ (0.0002)	−0.001^***^ (0.0001)
Constant	0.291	0.281
Fixed effect of time	P	P
Fixed effect of place	P	P
Sample capacity	1,800	1,800
R-squared	0.934	0.976

We have applied a logarithmic transformation to the variable, per capita gross regional product, in our model analysis. Y1 and Y2 separately represent the number of healthcare workers and village clinics in rural Sichuan. Standard errors are reported in parentheses. Significance at the 1% level are denoted by ***. The raw number of healthcare consultations in primary-level areas was converted to per 1,000 units.

### 3.3 Impact of the CCMSC policy on healthcare service utilization in village clinics

According to Table 5, first, the number of healthcare consultations in village clinics exhibited a statistically significant average increase of 0.089 (c. 3.7%) per 1,000 rural population in rural Sichuan after implementing the CCMSC policy. Second, the number of emergency consultations exhibited a statistically significant average increase of 0.082 (c. 3.5%) per 1,000 rural population in rural Sichuan. Thus, healthcare service utilization in village clinics was enhanced. The enhancement of healthcare service utilization in village clinics was primarily attributed to a rise in the number of healthcare consultations and service institutions in primary-level areas, while the per capita gross regional product did not exhibit a significant impact. However, the number of doctors and healthcare beds in primary-level areas and the rural population had statistically significant negative impacts on healthcare service utilization in village clinics. This reflects macro-environmental changes in the reform of current healthcare service system in China. Chen et al. (16) suggested that there exists a substitution effect between township centers and village clinics. The increased number of doctors and healthcare beds in primary-level areas attracted more rural residents to seek healthcare services in township health centers and county-level public hospitals (53), thereby leading to a decline in the number of healthcare and emergency consultations in village clinics. Furthermore, village clinics had previously assumed the important role of primary healthcare consultations and referrals for rural patient. With the continuous outflow of rural residents from rural to urban areas, inpatient demand primarily concentrated in these higher-level healthcare service institutions, thereby leading to a decline in the number of recorded healthcare and emergency consultations in village clinics.

**Table 5:** Impact of compact county medical service community policy on healthcare service utilization in village clinics.

Variables	Y3	Y4
DID	0.089^**^ (0.039)	0.082^**^ (0.038)
Per capita gross regional product	0.129^**^ (0.064)	0.141^**^ (0.063)
Doctors in primary-level areas	−0.168^***^ (0.028)	−0.167^***^ (0.027)
Healthcare consultations in primary-level areas	0.564^***^ (0.017)	0.536^***^ (0.017)
Healthcare service institutions in primary-level areas	0.349^***^ (0.065)	0.320^***^ (0.063)
Healthcare beds in primary-level areas	−0.105^***^ (0.028)	−0.096^***^ (0.027)
Rural population	−0.003^***^ (0.0003)	−0.004 (0.0003)
Constant	1.082	1.104
Fixed effect of time	P	P
Fixed effect of place	P	P
Sample capacity	1,800	1,800
R-squared	0.950	0.953

We have applied a logarithmic transformation to the variable, per capita gross regional product, in our model analysis. Y3 and Y4 separately represent the number of healthcare and emergency consultations in rural Sichuan. Standard errors are reported in parentheses. Significance at the 1, and 5% level are denoted by ***, and **, respectively. The raw number of healthcare consultations in primary-level areas and the raw number of healthcare and emergency consultations were converted to per 1,000 units.

### 3.4 Parallel trend test

A staggered DID approach was adopted in the empirical analysis to evaluate the causal impact of the CCMSC policy on healthcare resource allocation and healthcare service utilization in village clinics in rural Sichuan, contingent upon satisfying the parallel trends assumption (30, 55). Specifically, in the absence of the CCMSC policy, each indicator of healthcare resource allocation and healthcare service utilization in village clinics would have followed the same trend in 36 pilot counties and 144 non-piloted cities, conditional on covariates (56). We employed an event-study methodology to estimate the treatment effect in each year. Its model is expressed via Equation 2:

$$Y_{\mathrm{it}}=\alpha_{0}+\sum_{j\neq 0}\beta_{j}.D_{\mathrm{it}}^{j}+\sum_{h=1}^{\kappa }\gamma_{h}.X_{\mathrm{it}}^{h}+\lambda_{i}+\theta_{t}+\varepsilon_{\mathrm{it}}$$ (2)

In Equation 3, $D_{\mathrm{it}}^{j}$ represents a series of time dummy variables for periods j relative to the intervention period, taking the value of 1 if county i in year t received the policy intervention in period j ; otherwise, 0. $\beta_{j}$ represents the treatment effect of the policy in the period j between the treatment group and the control group at each year j . We omitted the −1 as the baseline period, and hence there were 12 periods in total. j > = 0 represents the periods following the CCMSC policy intervention, and j < 0 represents periods before the policy intervention. If β was nonsignificant during periods for which j < 0, this would indicate that the parallel trend assumptions hold (57).

**Figure 2:**
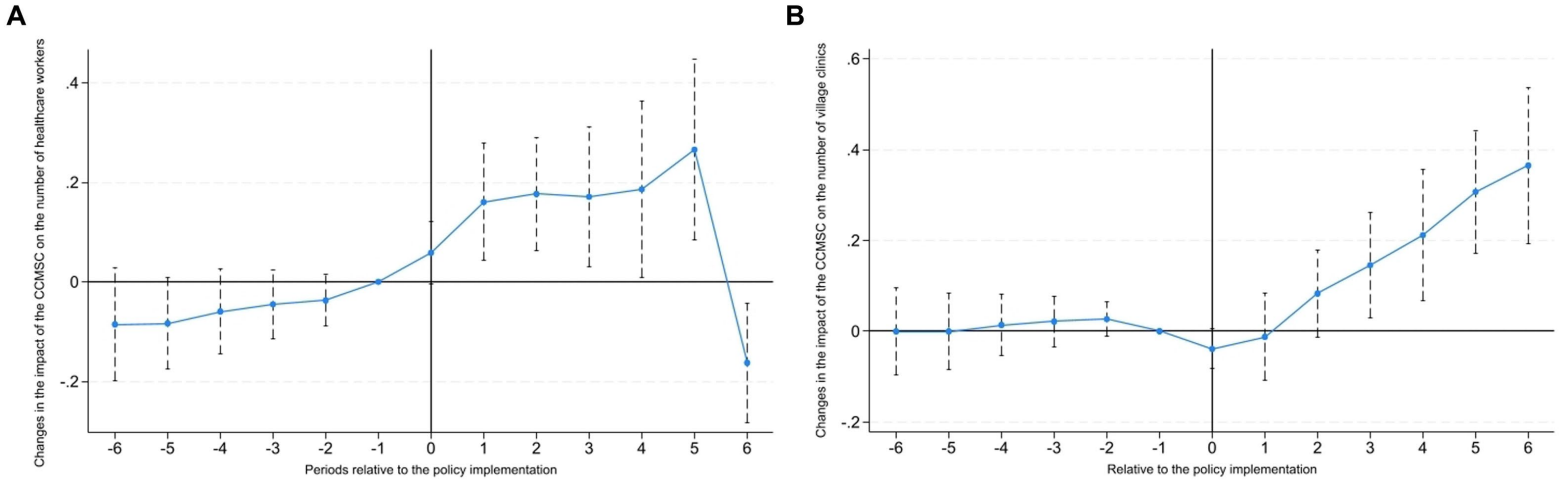
Parallel trend test for healthcare resource allocation in village clinics. The zero line represents the null hypothesis, indicating no significant change in healthcare resource allocation in village clinics: the number of healthcare workers **(A)** and village clinics **(B)**. Data points above or below the zero line reflect positive or negative changes, with the 95% confidence intervals showing statistical significance.

**Figure 3:**
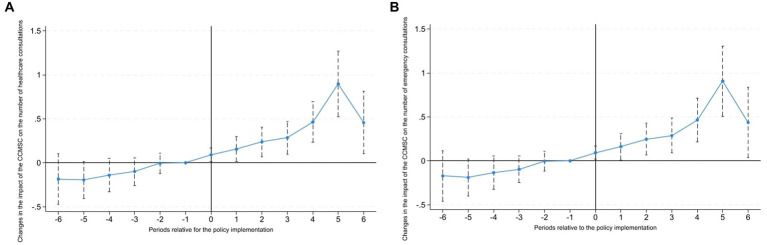
Parallel trend test for healthcare service utilization in village clinics. The zero line represents the null hypothesis, indicating no significant change in healthcare service utilization in village clinics: the number of healthcare consultations **(A)** and emergency consultations **(B)**. Data points above or below the zero line reflect positive or negative changes, with the 95% confidence intervals showing statistical significance.

Figures 2A,B, 3A,B separately show the dynamic impact of the CCMSC policy on healthcare resource allocation and healthcare service utilization in village clinics in rural Sichuan. Both figures illustrate nonsignificant policy pre-intervention trend differences between the treatment and control groups, as the 95% confidence intervals (CIs) of each indicator for the difference in trends include zero. This demonstrates the validity of the parallel trend assumption.

As shown in Figure 2A, the policy implementation results regarding healthcare resource allocation in the number of healthcare workers reflect a significant upward trend during the policy from 2020 to 2023. However, the impact of the CCMSC policy decreased dramatically from 2022 to 2023, albeit remaining significantly positive. This shift indicates that after the COVID-19 pandemic, demand for healthcare workers in village clinics reduced dramatically because rural residents were limited in their ability to seek health care in higher-level healthcare service institutions, such as township health centers and urban tertiary hospitals (14). In addition, Figure 2B shows that the impact of the CCMSC policy on the number of village clinics presented as an upward trend with a clear delay effect. A potential reason for this is that the construction of village clinics in some counties required time. These shifts indicate that the coordinated governance mechanism for the implementation of the CCMSC policy enhanced healthcare resource allocation in village clinics, which aligns with the policy’s goal of improving the quality of rural healthcare services.

As presented in Figures 3A,B, the trends in healthcare and emergency consultations in village clinics exhibited similar patterns, suggesting that, first, owing to the COVID-19 pandemic, the county-level public hospitals distributed healthcare resources to village clinics, thereby optimizing the healthcare resource allocation there; in contrast, during the COVID-19 period, rural residents could only seek local healthcare services in village clinics, which caused an increase in the number of healthcare and emergency consultation there. Note that after the pandemic, rural residents were free to seek health care anywhere, such as township health centers and urban tertiary hospitals, thereby leading to a decline in utilization of village clinics.

### 3.5 PSM DID

Although the list of 36 pilot counties for the implementation of the CCMSC policy was released officially after eliminating Junlian county, we acknowledge that the 36 counties were not selected randomly, which may have introduced potential selection bias and endogeneity. It is noted that the list of pilot counties for the CCMSC policy was formally released in 2019 (8). To identify the selection criteria for the 36 pilot counties and assess potential selection bias, we first estimated a logit regression model predicting pilot assignment based on pre-treatment county characteristics (58).

As presented in Supplementary Table S2, we found evidence of positive selection bias in terms of specific county attributes. Results indicated that per capita gross regional product and rural population were not statistically significant, indicating that the economic development level and population proportion were not critical factors in selecting a pilot county. Moreover, the number of doctors, healthcare service institutions, and beds in primary-level areas were also not statistically significant, confirming that the selection of pilot counties was not attributed to whether the county had abundant healthcare resources. However, the number of healthcare consultations in primary-level areas was statistically significant at the 1% level (coef. = 0.242), indicating that greater demand for healthcare services was the critical factor in selecting the pilot counties. Although demand for healthcare services in primary-level areas was the only selection criterion, minor selection bias nevertheless existed.

**Figure 4:**
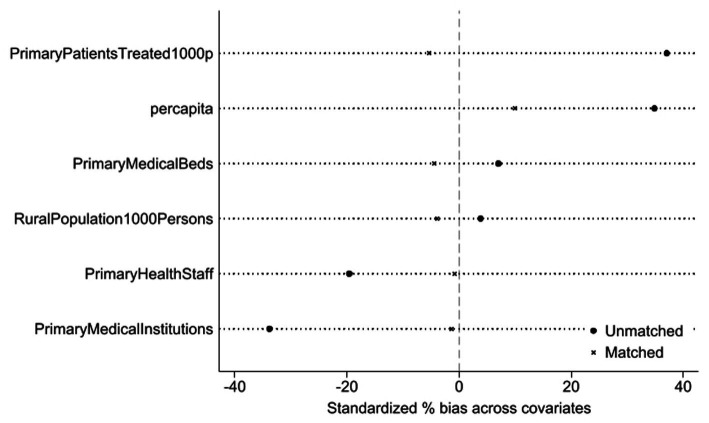
Results of the propensity score matching test.

To reduce the potential effect of selection bias, we adopted PSM-DID with two-way fixed effects to further evaluate the impact of CCMSC policy at different periods before and after the policy intervention to mitigate potential endogeneity (29, 59). Figure 4 and Table 1 present the results of the PSM using nearest neighbor matching with a caliper.

The matching process should lead to no significant differences between the treatment and control groups. As shown in Table 1, before matching, there were four variables with significant differences between groups, namely per capita gross regional product, healthcare service institutions, consultations, and doctors in primary-level areas. Following matching, there were 873 county-year observations in total, 178 in the treatment group and 695 in the control group. Absolute values of the standard deviation for the five variables were less than or equal to 10%; the control group was comparable to the treatment group with respect to these control variables (Table 1 and Figure 4). Moreover, between-groups t-test results for all control variables were not statistically significant following PSM. Therefore, these control variables were suitable for this research. After PSM, the control group was comparable to the treatment group with respect to the control variables. Our nearest neighbor matching estimations with caliper were reliable.

Tables 6, 7 separately present the results of PSM-DID for healthcare resource allocation and healthcare service utilization in village clinics after matching. The estimates indicate that implementing the CCMSC policy in rural Sichuan had a positive impact on healthcare resource allocation and healthcare service utilization in village clinics. Specifically, the number of healthcare workers and village clinics significantly increased by 0.163 (8.3%) and 0.089 (6.2%), respectively, per 1,000 rural residents. Additionally, the number of healthcare and emergency consultations in village clinics significantly increased by 0.164 (6.8%) and 0.159 (6.9%), respectively.

**Table 6:** Results for the impact of the CCMSC policy on healthcare resource allocation in village clinics after nearest neighbor (NN) matching with a caliper.

Variables	Y1	Y2
DID	0.163^***^ (0.056)	0.089^**^ (0.041)
Per capita gross regional product	0.125 (0.167)	0.023 (0.072)
Doctors in primary-level areas	0.273^***^ (0.063)	−0.047 (0.033)
Healthcare consultations in primary-level areas	−0.085^***^ (0.031)	−0.011 (0.020)
Healthcare service institutions in primary-level areas	0.897^***^ (0.144)	1.282^***^ (0.108)
Healthcare beds in primary-level areas	−0.075 (0.054)	−0.043 (0.033)
Rural population	−0.002^**^ (0.001)	−0.002^***^ (0.001)
Constant	0.755	0.736
Fixed effect of time	P	P
Fixed effect of place	P	P
Sample capacity	1,000	1,000
R-squared	0.935	0.949

The table presents estimates of the impact of the CCMSC policy on the allocation of healthcare resources in village clinics by a PSM-DID model with two-way fixed effects of county i and year t . We employed panel data covering the full period (2014–2023), weighted by the propensity scores estimated from the pre-policy period (2014–2018). The matching was 1:3 nearest neighbor matching with a caliper. After estimating the propensity scores, we set the caliper to 0.25 times the standard deviation of the propensity score (75). The standard deviation of the propensity score was 0.074. Hence, the caliper was 0.02. DID was a binary variable, which equalled 1 if the county i implemented the CCMSC policy in year t , otherwise it equalled 0. PSM-DID represents a binary variable after PSM, which equalled 1 if the county i implemented the CCMSC policy in year t , otherwise it equalled 0. Y1 and Y2 separately represent the number of healthcare workers and village clinics in rural Sichuan. Standard errors are reported in parentheses. Significance at the 1, and 5% level are denoted by ***, and **, respectively. We applied a logarithmic transformation to the variable, per capita gross regional product, in our model analysis. The raw number of healthcare consultations in primary-level areas was converted to per 1,000 units.

**Table 7:** Results for the impact of the CCMSC policy on healthcare service utilization in village clinics after nearest neighbor (NN) matching with a caliper.

Variables	Y3	Y4
DID	0.164^**^ (0.080)	0.159^**^ (0.078)
Per capita gross regional product	0.028 (0.223)	0.085 (0.213)
Doctors in primary-level areas	−0.130^**^ (0.064)	−0.123^**^ (0.061)
Healthcare consultations in primary-level areas	0.502^***^ (0.060)	0.480^***^ (0.058)
Healthcare service institutions in primary-level areas	0.445^**^ (0.198)	0.416^**^ (0.193)
Healthcare beds in primary-level areas	−0.189^***^ (0.064)	−0.184^***^ (0.063)
Rural population	−0.005^***^ (0.001)	−0.006^***^ (0.001)
Constant	1.845	1.806
Fixed effect of time	P	P
Fixed effect of place	P	P
Sample capacity	1,000	1,000
R-squared	0.952	0.951

The table presents estimates of the impact of the CCMSC policy on the utilization of healthcare services in village clinics using a PSM-DID model with two-way fixed effects of county i and year t . We employed panel data covering the full period (2014–2023), weighted by the propensity scores estimated from the pre-policy period (2014–2018). The matching was 1:3 nearest neighbor matching with a caliper. After estimating the propensity scores, we set the caliper to 0.25 times the standard deviation of the propensity score (75). The standard deviation of the propensity score was 0.074. Hence, the caliper was 0.02. DID was a binary variable, which equalled 1 if the county i implemented the CCMSC policy in year t , otherwise it equalled 0. PSM-DID represents a binary variable after PSM, which equalled 1 if the county i implemented the CCMSC policy in year t , otherwise it equalled 0. Y3 and Y4 separately represent the number of healthcare and emergency consultations in rural Sichuan. Standard errors are reported in parentheses. Significance at the 1, and 5% level are denoted by ***, and **, respectively. We applied a logarithmic transformation to the variable, per capita gross regional product, in our model analysis. The raw number of healthcare consultations in primary-level areas and the raw number of healthcare and emergency consultations were converted to per 1,000 units.

### 3.6 Parallel trend test after matching

**Figure 5:**
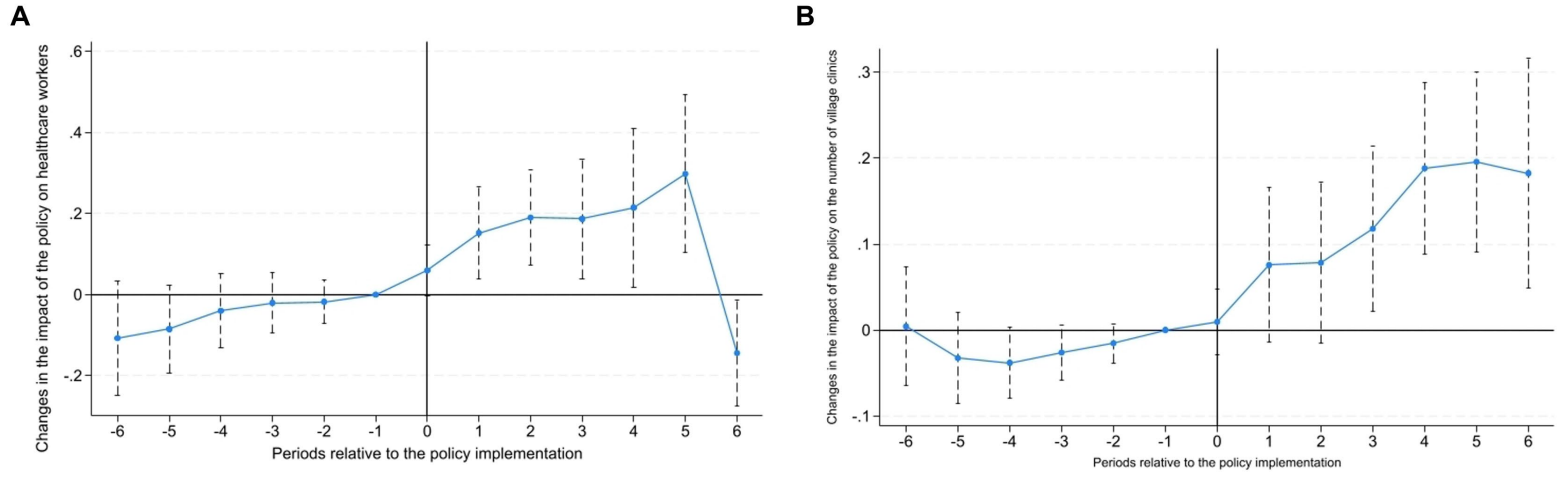
Results of the parallel trend test of healthcare resource allocation in village clinics after matching The zero line represents the null hypothesis, indicating no significant change in healthcare resource allocation in village clinics: the number of healthcare workers **(A)** and village clinics **(B)**. Data points above or below the zero line reflect positive or negative changes, with the 95% confidence intervals showing statistical significance.

**Figure 6:**
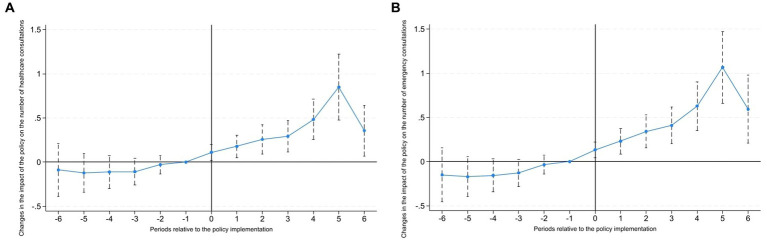
Results of the parallel trend test of healthcare service utilization in village clinics after matching. The zero line represents the null hypothesis, indicating no significant change in the healthcare service utilization in village clinics: the number of healthcare consultations **(A)** and emergency consultations **(B)**. Data points above or below the zero line reflect positive or negative changes, with the 95% confidence intervals showing statistical significance.

To further confirm the reliability and reduce the impact of potential selection bias, we conducted a parallel trends test after matching. As presented in Figures 5, 6, there were no significant pre-intervention trend differences between the treatment and control groups with the 95% CIs of each indicator of the difference in trends before implementation of the CCMSC policy including zero. This further demonstrates the validity of parallel trends assumption (55, 57). The model used in this test is as expressed in Equation 2:

As shown in Figure 5A, the number of healthcare workers in village clinics reflected a significant upward trend during the policy intervention period and the subsequent years. The trend was similar to its pre-matching trend. Additionally, Figure 5B shows that the impact of the CCMSC policy on the number of village clinics experienced an upward trend with a clear hysteresis, but after the COVID-19 pandemic, its impact began to reduce. The trend was different from its pre-matching trend. A potential reason is also the lockdown due to the COVID-19 pandemic. The construction of village clinics in some counties was forced to be paused, leading to the hysteresis.

As presented in Figures 3A,B, the trends in healthcare and emergency consultations in village clinics exhibited similar patterns mirroring their pre-matching trends. This suggested that with the optimization of healthcare resources in village clinics, the proportion of healthcare consultations for rural residents increased. Meanwhile, during the COVID-19 pandemic (2020–2022), there was an increase in the demand for emergency consultations when individuals experienced COVID symptoms. In addition, owing to the pandemic lockdown, individuals could only seek treatment in village clinics, thereby leading to an increase in the number of healthcare and emergency consultations. After the COVID-19 pandemic (2023), rural residents were no longer restricted by the lockdown and could freely access healthcare services at higher-level healthcare service institutions (14); consequently, healthcare service utilization in village clinics (i.e., the number of healthcare and emergency consultations) exhibited a downward trend.

### 3.7 Robustness tests

After matching, we tested the robustness of our results using three methods. First, we conducted placebo tests to exclude the possibility that our estimated results were attributable to random factors or the influence of unobservable confounding factors (60, 61). These results are presented in Supplementary Figures S1A,B for healthcare resource allocation and Supplementary Figures S2A,B for healthcare service utilization in village clinics. A placebo test was employed for each indicator of healthcare resource allocation and healthcare service utilization in village clinics in rural Sichuan. We utilized Stata 18 software to establish the dummy interaction term and randomly selected 36 pilot sites to form a pseudo-treatment group. Specifically, we performed regressions for the pseudo-treatment group and repeated this action 500 times, thereby producing 500 coefficients and *p*-values for all indicators of healthcare resource allocation and healthcare service utilization in village clinics. Results indicated that all estimated coefficients for healthcare resource allocation and healthcare service utilization in village clinics were greater than their true values and followed a normal distribution, with most of the p-values of all indicators surpassing 0.1. This suggests that our original significant DID results were not obtained randomly; that is, they were reliable.

The study period (2014–2023) overlapped the COVID-19 pandemic (2020–2022). Considering the disruptions caused by the COVID-19 pandemic, we further estimated whether the CCMSC policy impact persisted independent of the pandemic lockdowns via two sensitivity analyses. First, as presented in Supplementary Table S3, the number of healthcare workers and village clinics continued to significantly increase after excluding years 2020–2022 (62). Moreover, Supplementary Table S4 illustrates that there were also significant increases in the number of healthcare and emergency consultations in village clinics. This confirms that implementing the CCMSC policy significantly enhanced healthcare resource allocation in village clinics during the COVID-19 pandemic (62).

Second, to isolate policy effects from COVID-19 disruptions (2020–2022), we assessed the interaction between the treatment variable and the dummy variable indicating the COVID-19 pandemic period (2020–2022), thereby estimating whether COVID-19 significantly affected the impact of the CCMSC policy (Equation 3) (63).

$$\begin{array}{c} Y_{\mathrm{it}}=\alpha +\beta .\text{CCMS}C_{\mathrm{it}}+\beta_{1}.\text{CCMS}C_{\mathrm{it}}\times \text{pandemic}\\ +\sum_{h=1}^{\kappa }\gamma_{h}.X_{\mathrm{it}}^{h}+\lambda_{i}+\theta_{t}+\varepsilon_{\mathrm{it}} \end{array}$$ (3)

The coefficient $\beta_{1}$ reflects the additional impact of the COVID-19 pandemic on the treatment group. The variable pandemic is a binary indicator that equals 1 for years 2020–2022 and 0 otherwise.

Results demonstrated that the estimated impacts of the CCMSC policy on healthcare resource allocation (Supplementary Table S5) and healthcare service utilization (Supplementary Table S6) in village clinics in the 3 years following the implementation of the policy were statistically significant. Note that the causal impact of the CCMSC policy on both indicators of healthcare resource allocation may be influenced by the COVID-19 pandemic between 2020 and 2022. Nevertheless, the regressions for the impact of the CCMSC policy on the number of village clinics and healthcare workers remained significantly positive. Therefore, the sensitivity analysis supported our inference that the impact of the CCMSC policy persisted independent of the pandemic lockdowns.

Third, we lagged the key dependent variable (DID) to alleviate the serial autocorrelation problem arising from the highly persistent nature of healthcare resource allocation and healthcare service utilization in village clinics. This controlled for the possible interference of such unobservable individual characteristics that evolve over time on the estimation results (64). The results utilizing the one-period lag of the key dependent variable (L. DID) are presented separately in Supplementary Tables S7,8 to address potential endogeneity concerns. As presented in Supplementary Table S7, the coefficients of the L. DID term for each indicator of healthcare resource allocation remained positive and statistically significant at the 1% level (coef. = 0.121 for the number of healthcare workers; coef. = 0.048 for the number of village clinics). As presented in Supplementary Table S8, the coefficients of the L. DID term for each indicator of healthcare service utilization in village clinics remained positive and statistically significant at the 5% level (coef. = 0.070 for the number of healthcare consultation; coef. = 0.076 for the number of emergency consultation). This further confirmed the reliability for our original results.

## 4 Discussion

Our study is the first to explore the causal impact of the CCMSC policy on healthcare resource allocation (the number of healthcare workers and village clinics) and healthcare service utilization (the number of healthcare and emergency consultations) in village clinics in rural Sichuan; this is a critical area of current healthcare service system reform. Our findings demonstrated that after matching, implementation of the CCMSC policy was effective for improving healthcare resource allocation and healthcare service utilization in village clinics alongside implementation time. Specifically, following matching, the observed average increase of 0.163 (8.3%) healthcare workers and 0.089 (6.2%) village clinics per 1,000 rural population in the in the pilot counties indicates a significant impact of the CCMSC policy. Moreover, the number of healthcare and emergency consultations in village clinics separately increased by 0.164 (6.8%) and 0.159 (6.9%) per 1,000 rural population, respectively. Additionally, the key factors in implementing the CCMSC policy were found to be township health centers, the COVID-19 pandemic (2020–2022), economic level, and the number of doctors in primary-level areas. Hence, there are three main aspects of our research that we further discuss here.

First, the key finding of our study was that implementing the CCMSC policy was effective for enhancing healthcare resource allocation and healthcare service utilization in village clinics. This finding differs from that of an earlier study conducted in primary Sichuan (9). Ding and Zhou (9) found that the impact of the CCMSC policy was statistically nonsignificant for the allocation of healthcare human resources and facilities in pilot counties in Sichuan. Effective implementation of the CCMSC policy helps establish a coordinated governance mechanism for healthcare resource allocation from county-level public hospitals as the lead to township health centers as the central hub and village clinics as the foundation (10, 65). The CCMSC policy should be a comprehensive policy combination system. The potential reasons that explain the positive impact on healthcare resource allocation and healthcare service utilization are the combination of economic incentives, coordinated governance, balance between supply and demand, along with other mechanisms. For instance, the number of doctors in village clinics increased (23) and the number of healthcare consultations for rural residents in village clinics also increased (66). We also found that the effect of the CCMSC policy increased over time. Hence, the Sichuan government should continue to implement the CCMSC policy, integrate healthcare resources based on local residents’ healthcare demands, and return surplus resources (e.g., capital) to the county-level public hospitals for unified management.

Additionally, the CCMSC policy aims to promote healthcare resource integration and enhance healthcare resource allocation and healthcare service utilization in primary-level areas, especially in rural areas. However, the sustainable optimization of healthcare resource allocation in higher-tier healthcare service institutions (county-level public hospitals and township health centers) may draw rural patients away from village clinics, thereby causing a “siphon effect.” Specifically, there remains a significant discrepancy in healthcare service delivery capacity and healthcare technology between county-level public hospitals and rural healthcare service institutions (e.g., village clinics) (1). When the implementation of the CCMSC policy significantly enhanced healthcare resource allocation, such as hardware and specialists, to county-level public hospitals and township health centers, the disparity in perceived healthcare service quality widened. This enabled rural residents to bypass village clinics and seek healthcare services at higher-tier healthcare service institutions, even residents with only minor illnesses (1, 67, 68). Furthermore, those higher-tier healthcare service institutions focus on their own operational revenue and bed occupancy rates, and were unlikely to reject patients, thereby absorbing the patient pool that should theoretically be served at village clinics (69).

To mitigate this internal competition and to rebalance patient flow at different-tier healthcare service institutions, county-level public hospitals as the lead for the implementation of CCMSC policy should establish clearer functional roles with township health centers and village clinics, and implement robust two-way referral mechanisms. Coordinated governance through administrative unification is insufficient to align the divergent interests of healthcare service institutions of different tiers. Hence, the implementation of CCMSC policy should regulate the scope of healthcare service deliveries among county-level public hospitals, township health centers, and village clinics based on the types of diseases. For instance, village clinics could be responsible for delivering primary healthcare for patients and facilitate referrals for some patients with serious diseases to higher-tier healthcare service institutions (i.e., township health centers and county-level public hospitals) (5, 53). Meanwhile, village clinics should also improve their capabilities to treat chronic disease; this would enable rural patients with minor chronic diseases and symptoms to seek health care there directly, making it unnecessary to visit higher-tier healthcare service institutions. This would both enhance the accessibility of healthcare and reduce the costs of transportation and treatment for patients. This could reduce the impact of internal competition among different tiers and motivate rural patients to utilize rural healthcare services, thereby addressing the problem of drawing patients away from village clinics.

Second, we found an impact of the CCMSC policy on healthcare service utilization in village clinics, in contrast to a previous study (16). While Chen et al. (16) indicated a 14.6 percentage-point decrease in village clinic utilization, we observed significant increases in both healthcare and emergency consultations (6.8 and 6.9%, respectively) following implementation of the CCMSC policy. This suggests a reversal of the previously declining trend in village clinic utilization. However, as highlighted in Table 5, the number of doctors and beds in primary-level areas had statistically significant negative impacts on village clinic utilization, confirming a substitution effect. Specifically, implementation of the CCMSC policy improved the healthcare resource allocation of not only village clinics but also township health centers. The township health centers as the central hubs for the implementation of the CCMSC policy possess more abundant healthcare resources than village clinics (9, 10). This naturally led rural residents to seek higher-quality healthcare services and bypass village clinics. Despite this substitution effect, the net impact of the CCMSC policy remained positive due to vertical complementarity. As presented in Table 6, the number of healthcare workers in village clinics increased significantly after matching. Implementation of the CCMSC policy fundamentally optimized healthcare resource allocation in village clinics, which can be attributed to a coordinated governance mechanism. High-quality healthcare resources are serially allocated to township health centers and village clinics. By addressing the shortage of healthcare resources in village clinics, implementation of the CCMSC policy improved the quality of healthcare services in such clinics. This likely improved the trust of rural residents in the healthcare capacity of village clinics and encouraged them to utilize these clinics to treat common illnesses. Therefore, while the optimization of healthcare resource allocation in township health centers inevitably led some rural patients to seek care there, healthcare resource allocation in village clinics is continuously improving. Crucially, village clinics were enhancing the capacity to provide high-quality services, fostering complementarity with township health centers. This positive policy effect explains the net positive utilization rates observed, suggesting that the CCMSC policy currently functions more as a healthcare capacity-strengthening intervention than merely as competition-inducing.

There are several possible explanations for the increase in village clinic utilization. On the supply side, after implementing the CCMSC policy, the county-level public hospitals took the lead in uniting township health centers and village clinics as a community of shared interests (10). The implementation of CCMSC policy focused on coordinated governance and unified management of healthcare resources, including human resources, finance, facilities, drugs, and service quality standardization (9). Based on coordinated governance and unified management, unified purchase and allocation were carried out, and township health centers and village clinics did not need to purchase costly medical equipment and were allocated sufficient healthcare workers on demand. This addressed the issue of high-quality healthcare resources being highly concentrated in urban tertiary hospitals (53). Furthermore, with the unified management and allocation of healthcare resources, the number of healthcare workers in rural areas has increased (36) and has gradually met public expectations of primary care facilities (70).

Additionally, it is important to note that there was an observed significant growth with a clear hysteresis in the number of village clinics after matching. However, after the COVID-19 pandemic, the policy’s effect reduced but remained positive as depicted in Figure 5B, suggesting potential saturation in physical infrastructure development. This trend has important implications for future policy and adjustments within the context of the CCMSC initiative. The diminishing rate of increase in the number of village clinics may indicate that the such clinics should focus on improving the quality and efficiency of healthcare services in subsequent years, instead of focusing on further rapid expansion of village clinic density. Hence, through the coordinated governance mechanism, county-level public hospitals should distribute high-quality healthcare resources to village clinics, including doctors, healthcare beds, finance, healthcare equipment, and the integration of telemedicine technologies (37, 46, 54, 71). The optimization of healthcare resources could further enhance the healthcare service capacities of village clinics, thereby enhancing their service efficiency. In addition, county-level public hospitals could distribute healthcare experts to provide training for healthcare workers in village clinics, thereby improving the healthcare service capacity and efficiency of such clinics (17). Ultimately, these strategic adjustments could not only promote the sustainable development of the rural healthcare system but also contribute to achieving the original goal of delivering equitable and high-quality healthcare services to all residents in Sichuan Province.

On the demand side, several studies indicated that most rural residents did not trust rural healthcare services and were not satisfied with the quality of rural healthcare services (53). With the optimization of healthcare resource allocation in rural areas, rural residents can utilize village clinics for treating common and chronic diseases, which improves the accessibility of health care, reduces transportation and time costs, and enhances the willingness of the populace to utilize village clinics (5, 72, 73). When rural patients experience symptoms, they are no longer required to travel long distances to urban tertiary hospitals and county-level public hospitals.

Third, it should be noted that the study period (2014–2023) overlapped the COVID-19 pandemic (2020–2022), which may have confounded the observed increases in healthcare and emergency consultations in village clinics. Pandemic-related travel restrictions and lockdowns may have forced rural residents to use village clinics as the only accessible care option, resulting in a temporary forced substitution effect. To mitigate this confounding bias, we conducted two sensitivity analyses: introducing a CCMSC–pandemic interaction term and excluding the 2020–2022 period (62, 63). Both analyses confirmed that the positive effects of the CCMSC policy remained robust and statistically significant, indicating that the policy improvements were not driven solely by COVID-19 restrictions.

To check whether the impact of CCMSC policy persisted independent of the COVID-19 pandemic limitations and lockdowns, we employed a sensitivity analysis by excluding the pandemic period (2020–2022) and then conducted regressions to test whether the policy impact persisted independent of the pandemic lockdowns. As presented in Supplementary Table S4, the number of healthcare workers and village clinics remained significantly increased after excluding years 2020–2022. Moreover, Supplementary Table S5 shows that there were also increases in the number of healthcare and emergency consultations in village clinics. However, the number of doctors in primary-level areas had significantly negative impacts on healthcare service utilization. Residents with low trust in the quality of healthcare services in village clinics had a high likelihood of seeking healthcare services in county-level public hospitals and urban tertiary hospitals when the local lockdown was lifted. Results indicated that during the COVID-19 pandemic (2020–2022), the positive impact of the CCMSC policy on healthcare resource allocation and healthcare service utilization in village clinics remained statistically significant. This further confirms that the impact of CCMSC policy persisted independent of the pandemic lockdowns.

Although this study provides valuable insights, we acknowledge its limitations. First, a key limitation is that pilot counties may not be randomly selected, which may introduce selection bias and endogeneity. Although we used PSM, two-way fixed effects, and parallel trend tests to reduce confounding, we cannot fully exclude the possibility that pre-existing advantages of pilot areas may have slightly amplified the estimated policy effects. This should be considered when interpreting the causal impacts of the CCMSC policy. Second, there were potentially unobserved confounders. For instance, new village clinics in a pilot location may change how far local residents will have to travel to access healthcare and may also influence their social interactions. However, the place of residence of patients is private information that was unavailable to us. Hence, we did not consider the distance variable for ethical reasons. Third, the study period (2020–2022) coincided with the COVID-19 pandemic, which caused systemic disruptions to China’s primary healthcare system. Although we conducted sensitivity analysis to verify the consistency of the policy effect estimates, our analysis could not fully control for this exogenous shock, which may potentially confound the estimated policy effects. Finally, owing the limitation of data concerning telemedicine, we could not estimate the impact of telemedicine expansion on the effect of the CCMSC policy, although the expansion may have confounded the estimated policy effects.

## 5 Conclusion

The findings of this study have important implications for healthcare policy and practice. In the Chinese context, the implementation of the CCMSC policy is associated with the establishment of a coordinated governance mechanism for healthcare resource allocation, encompassing the deployment of healthcare workers and financial support. Through this mechanism, county-level public hospitals, township health centers, and village clinics can operate as an integrated unit. Specifically, county-level public hospitals take the lead and are responsible for unified purchasing and distributing resources to rural areas; township health centers act as the central hub, and village clinics form the foundation.

This contributes to unified management and operation for healthcare resource allocation from county-level public hospitals to village clinics and forms a mode for sharing healthcare resources. With the optimization of healthcare resource allocation in rural areas, village clinics do not need to independently purchase additional healthcare resources, and rural residents can seek high-quality healthcare services nearby. However, while this shift in utilization reflects improved accessibility and suggests progress toward service equity, it is necessary to acknowledge that this change may primarily represent a shift in the location of care driven by resource availability. Further research is required to determine whether these structural changes translate into improved health outcomes beyond mere utilization statistics.

Implementation of the CCMSC policy alongside the coordinated governance mechanism may provide references for other counties in China and developing countries without a compulsory gatekeeping system. From a government perspective, this study provides empirical evidence that the coordinated governance mechanism is a vital health policy associated with optimized healthcare resource allocation in rural areas and improved rural residents’ trust in the quality of healthcare services provided by village clinics. Initiatives could be adopted to further optimize the two-way referral system for rural patients between county-level public hospitals and rural healthcare service institutions (township health centers and village clinics) and further improve the efficiency of disease treatment in rural areas to facilitate the implementation of the CCMSC policy.

For policymakers, the substantial increase in healthcare resource input and related costs following the CCMSC policy indicates that cautious monitoring and management are required to guarantee long-term effectiveness and sustainability. It is crucial for policymakers to ensure that these increased inputs are supported by sustainable funding mechanisms instead of relying solely on temporary subsidies, to maintain the sustainability of the policy’s impact. Furthermore, the government should publicize healthcare services in village clinics, by means of deploying healthcare workers and the regular provision of voluntary healthcare services. Overall, it is essential to ensure the continuous implementation and refinement of the CCMSC policy.

## Data Availability

The datasets presented in this study can be found in online repositories. The names of the repository/repositories and accession number(s) can be found: data were collected from the Sichuan Health Statistics Yearbooks and Sichuan Statistical Yearbooks between 2014 and 2022. The link for the Sichuan Health Statistics Yearbooks is https://wsjkw.sc.gov.cn/scwsjkw/njgb/tygl.shtml. The link for the Sichuan Statistical Yearbooks is https://tjj.sc.gov.cn/scstjj/c105855/nj.shtml.
