# Supplementary material for: Impact of the compact county medical service community policy on healthcare resource allocation and healthcare service utilization in village clinics: a difference-in-differences analysis in rural Sichuan, China
Source: Front Public Health. 2026 Jun 1;14:1826414. doi: 10.3389/fpubh.2026.1826414 (PMC13265455; doi:10.3389/fpubh.2026.1826414)

***Supplementary Material***

**1. Supplementary Tables**

**Supplementary Table 1**. List of piloted counties implementing the compact county medical service community policy in Sichuan Province

| **37 Piloted Counties** | | | | | |
| --- | --- | --- | --- | --- | --- |
| **City** | **Number** | **List of Counties** | **City** | **Number** | **List of Counties** |
| Chengdu | 5 | Xindu  (2020) | Leshan | 1 | Muchuan  (2019) |
|  |  | Qingbaijiang  (2019) | Nanchong | 1 | Shunqing  (2020) |
|  |  | Qionglai  (2020) | Yibing | 3 | Xingwen  (2019) |
|  |  | Xinjin  (2019) |  |  | Jiang'an  (2019) |
|  |  | Pujiang  (2020) |  |  | Junlian  (2023) |
| Zigong | 2 | Gongjing  (2019) | Guang’an | 2 | Guang'an  (2020) |
|  |  | Ziliujing  (2019) |  |  | Huaying  (2020) |
| Panzhihua | 2 | Miyi  (2018) | Dazhou | 1 | Tongchuan  (2019) |
|  |  | Yanbian  (2019) | Bazhou | 1 | Pingchang  (2019) |
| Luzhou | 2 | Lu  (2020) | Ya’an | 1 | Shimian  (2019) |
|  |  | Hejiang  (2020) | Meishan | 1 | Qingshen  (2019) |
| Deyang | 4 | Mianzhu  (2019) | Ziyang | 1 | Anyue  (2020) |
|  |  | Guanghan  (2020) | Aba | 2 | Wenchuan  (2019) |
|  |  | Zhongjiang  (2019) |  |  | Jiuzhaigou  (2020) |
|  |  | Luojiang (2017) | Ganzi | 2 | Ganzixian  (2019) |
| Mianyang | 1 | Pingwu  (2020) |  |  | Seda  (2019) |
| Guangyuan | 1 | Chaotian  (2019) | Liangshan | 2 | Xichang  (2018) |
| Suining | 1 | Anju  (2019) |  |  | Dechang  (2018) |
| Neijiang | 1 | Longchang  (2020) | Total | 37 Counties | |

**Note:** The implementation year for each pilot county has been reported in parentheses.

**Supplementary Table 2.**

**Table S2.** Results for logit regression

| **Variables** | **Probability to be treated** |
| --- | --- |
| **Per capita gross regional product** | 0.237  (0.214) |
| **Doctors in primary-level areas** | -0.141  (0.190) |
| **Healthcare consultations in primary-level areas** | 0.242^***^  (0.081) |
| **Healthcare service institutions in primary-level areas** | 0.224  (0.301) |
| **Healthcare beds in primary-level areas** | -0.085  (0.168) |
| **Rural population** | -0.001  (0.001) |
| **Constant** | -1.685 |
| **Sample capacity** | 900 |

**Note:** This Table reports the first stage of PSM. We have covered a logarithmic transformation for the variable, per capita gross regional product, in our model analysis. Standard errors have been reported in parentheses. The significance at the 1% level is denoted by ***. The raw number of healthcare consultations in primary-level areas was converted to per 1,000 units.

**Supplementary Table 3.**

**Table S3.** Sensitivity analysis for the impact of CCMSC policy on healthcare resource allocation in village clinics by excluding the COVID-19 pandemic periods (2020-2022) after matching

| **Variables** | **Y1** | **Y2** |
| --- | --- | --- |
| **DID** | 0.146^***^  (0.054) | 0.070^**^  (0.034) |
| **Per capita gross regional product** | 0.170  (0.111) | 0.034  (0.065) |
| **Doctors in primary-level areas** | 0.285^***^  (0.070) | -0.065^**^  (0.027) |
| **Healthcare consultations in primary-level areas** | -0.063^**^  (0.028) | -0.009  (0.016) |
| **Healthcare service institutions in primary-level areas** | 0.818^***^  (0.170) | 1.179^***^  (0.118) |
| **Healthcare beds in primary-level areas** | -0.027  (0.047) | -0.011  (0.023) |
| **Rural population** | -0.002  (0.001) | -0.002^***^  (0.001) |
| **Constant** | 0.502 | 0.761 |
| **Fixed effect of time** | P | P |
| **Fixed effect of place** | P | P |
| **Sample capacity** | 700 | 700 |
| **R-squared** | 0.944 | 0.960 |

**Note:** We have covered a logarithmic transformation for the variable, per capita gross regional product, in our model analysis. Y1 and Y2 separately represent the number of healthcare workers and village clinics in rural Sichuan. Standard errors have been reported in parentheses. The significance at the 1% and 5% level are separately denoted by *** and **. The raw number of healthcare consultations in primary-level areas was converted to per 1,000 units.

**Supplementary Table 4.**

**Table S4**. Sensitivity analysis for the impact of CCMSC policy on healthcare service utilization in village clinics by excluding the COVID-19 pandemic periods (2020-2022) after matching

| **Variables** | **Y3** | **Y4** |
| --- | --- | --- |
| **DID** | 0.155^**^  (0.076) | 0.150^**^  (0.074) |
| **Per capita gross regional product** | 0.006  (0.206) | 0.073  (0.196) |
| **Doctors in primary-level areas** | -0.150^**^  (0.067) | -0.143^**^  (0.063) |
| **Healthcare consultations in primary-level areas** | 0.524^***^  (0.063) | 0.502^***^  (0.061) |
| **Healthcare service institutions in primary-level areas** | 0.252  (0.211) | 0.263  (0.204) |
| **Healthcare beds in primary-level areas** | -0.103  (0.068) | -0.089  (0.066) |
| **Rural population** | -0.004^***^  (0.001) | -0.004^***^  (0.001) |
| **Constant** | 1.705 | 1.634 |
| **Fixed effect of time** | P | P |
| **Fixed effect of place** | P | P |
| **Sample capacity** | 700 | 700 |
| **R-squared** | 0.957 | 0.955 |

**Note:** We have covered a logarithmic transformation for the variable, per capita gross regional product, in our model analysis. Y3 and Y4 separately represent the number of healthcare and emergency consultations in rural Sichuan. Standard errors have been reported in parentheses. The significance at the 1% and 5% level are separately denoted by *** and **. The raw number of healthcare consultations in primary-level areas and the raw number of healthcare and emergency consultations were converted to per 1,000 units.

**Supplementary Table 5.**

**Table S5.** Sensitivity analysis for the impact of CCMSC policy on healthcare resource allocation in village clinics by interacting DID with the COVID-19 pandemic (2020-2022) after matching

| **Variables** | **Y1** | **Y2** |
| --- | --- | --- |
| **DID** | 0.109^**^  (0.053) | 0.096^**^  (0.037) |
| **Per capita gross regional product** | 0.131  (0.105) | 0.011  (0.072) |
| **Doctors in primary-level areas** | 0.220^***^  (0.054) | -0.051  (0.032) |
| **Healthcare consultations in primary-level areas** | -0.069^**^  (0.030) | -0.006  (0.021) |
| **Healthcare service institutions in primary-level areas** | 0.836^***^  (0.147) | 1.239^***^  (0.106) |
| **Healthcare beds in primary-level areas** | -0.024  (0.039) | -0.022  (0.028) |
| **Rural population** | -0.001  (0.001) | -0.002^***^  (0.001) |
| **COVID-19** | 0.088^**^ | 0.055^**^ |
|  | (0.039) | (0.027) |
| **Constant** | 0.574 | 0.796 |
| **Fixed effect of time** | P | P |
| **Fixed effect of place** | P | P |
| **Sample capacity** | 1,000 | 1,000 |
| **R-squared** | 0.926 | 0.945 |

We have covered a logarithmic transformation for the variable, per capita gross regional product, in our model analysis. Y1 and Y2 separately represent the number of healthcare workers and village clinics in rural Sichuan. Standard errors have been reported in parentheses. The significance at the 1% and 5% level are separately denoted by *** and **. The raw number of healthcare consultations in primary-level areas was converted to per 1,000 units.

**Supplementary Table 6.**

**Table S6.** Sensitivity analysis for the impact of CCMSC policy on healthcare service utilization in village clinics by interacting DID with the COVID-19 pandemic (2020-2022) after matching

| **Variables** | **Y3** | **Y4** |
| --- | --- | --- |
| **DID** | 0.163^**^  (0.080) | 0.153^**^  (0.075) |
| **Per capita gross regional product** | 0.008  (0.223) | 0.043  (0.221) |
| **Doctors in primary-level areas** | -0.136^**^  (0.068) | -0.140^**^  (0.066) |
| **Healthcare consultations in primary-level areas** | 0.509^***^  (0.060) | 0.493^***^  (0.059) |
| **Healthcare service institutions in primary-level areas** | 0.392^**^  (0.176) | 0.388^**^  (0.170) |
| **Healthcare beds in primary-level areas** | -0.186^***^  (0.064) | -0.171^***^  (0.062) |
| **Rural population** | -0.006^***^  (0.001) | -0.006^***^  (0.001) |
| **COVID-19** | -0.005 | -0.006 |
|  | (0.054) | (0.052) |
| **Constant** | 1.969 | 1.975 |
| **Fixed effect of time** | P | P |
| **Fixed effect of place** | P | P |
| **Sample capacity** | 1,000 | 1,000 |
| **R-squared** | 0.952 | 0.948 |

**Note:** We have covered a logarithmic transformation for the variable, per capita gross regional product, in our model analysis. Y3 and Y4 separately represent the number of healthcare and emergency consultations in rural Sichuan. Standard errors have been reported in parentheses. The significance at the 1% and 5% level are separately denoted by *** and **. The raw number of healthcare consultations in primary-level areas and the raw number of healthcare and emergency consultations were converted to per 1,000 units.

**Supplementary Table 7**

**Table S7.** Main results for the impact of the CCMSC policy on healthcare resource allocation in village clinics after lagging one period for the key independent variable

| **Variables** | **Y1** | **Y2** |
| --- | --- | --- |
| **L.DID** | 0.162^***^  (0.058) | 0.093^**^  (0.046) |
| **Per capita gross regional product** | 0.095  (0.101) | -0.005  (0.069) |
| **Doctors in primary-level areas** | 0.241^***^  (0.062) | -0.055  (0.034) |
| **Healthcare consultations in primary-level areas** | -0.088^***^  (0.031) | -0.013  (0.020) |
| **Healthcare service institutions in primary-level areas** | 0.992^***^  (0.137) | 1.313^***^  (0.102) |
| **Healthcare beds in primary-level areas** | -0.084  (0.056) | -0.044  (0.033) |
| **Rural population** | -0.003^**^  (0.001) | -0.002^***^  (0.001) |
| **Constant** | 0.844 | 0.806 |
| **Fixed effect of time** | P | P |
| **Fixed effect of place** | P | P |
| **Sample capacity** | 900 | 900 |
| **R-squared** | 0.941 | 0.951 |

**Note:** The table displays the results of a robustness test for the impact of CCMSC policy on healthcare resource allocation in village clinics by the lagged key dependent variable approach. DID is a binary variable, which equals 1 if county $i$ implemented the CCMSC policy in the year $t$, otherwise it equals 0. L.DID is a binary variable and represents the lagged treatment indicator, which equals 1 if county $i$ implemented the CCMSC policy in the year $t$, otherwise it equals 0. The coefficient on the L.DID captures the persistence of the key dependent variable over time. Standard errors have been reported in parentheses. Y1 and Y2 separately represent the number of healthcare workers and village clinics in rural Sichuan. Standard errors have been reported in parentheses. The significance at the 1% and 5% level are separately denoted by *** and **. We have covered a logarithmic transformation for the variable, per capita gross regional product, in our model analysis. The raw number of healthcare consultations in primary-level areas was converted to per 1,000 units.

**Supplementary Table 8**

**Table S8.** Main results for the impact of the CCMSC policy on healthcare service utilization in village clinics after lagging one period for the key independent variable

| **Variables** | **Y3** | **Y4** |
| --- | --- | --- |
| **L.DID** | 0.154^**^  (0.075) | 0.151^**^  (0.072) |
| **Per capita gross regional product** | 0.067  (0.218) | 0.119  (0.207) |
| **Doctors in primary-level areas** | -0.173^***^  (0.064) | -0.161^***^  (0.060) |
| **Healthcare consultations in primary-level areas** | 0.477^***^  (0.058) | 0.457^***^  (0.057) |
| **Healthcare service institutions in primary-level areas** | 0.537^***^  (0.159) | 0.497^***^  (0.154) |
| **Healthcare beds in primary-level areas** | -0.198^***^  (0.063) | -0.195^***^  (0.062) |
| **Rural population** | -0.006^***^  (0.001) | -0.006^***^  (0.001) |
| **Constant** | 1.908 | 1.844 |
| **Fixed effect of time** | P | P |
| **Fixed effect of place** | P | P |
| **Sample capacity** | 900 | 900 |
| **R-squared** | 0.958 | 0.957 |

**Note:** The table displays the results of a robustness test for the impact of CCMSC policy on healthcare resource allocation in village clinics by the lagged key dependent variable approach. DID is a binary variable, which equals 1 if county $i$ implemented the CCMSC policy in the year $t$, otherwise it equals 0. L.DID is a binary variable and represents the lagged treatment indicator, which equals 1 if county $i$ implemented the CCMSC policy in the year $t$, otherwise it equals 0. The coefficient on the L.DID captures the persistence of the key dependent variable over time. Standard errors have been reported in parentheses. Y3 and Y4 separately represent the number of healthcare and emergency consultations in rural Sichuan. Standard errors have been reported in parentheses. The significance at the 1% and 5% level are separately denoted by *** and **. We have covered a logarithmic transformation for the variable, per capita gross regional product, in our model analysis. The raw number of healthcare consultations in primary-level areas was converted to per 1,000 units.

1. **Supplementary Figures**

**Supplementary Figure 1.** The results of the placebo test for the impact of the CCMSC policy on healthcare resource allocation in village clinics


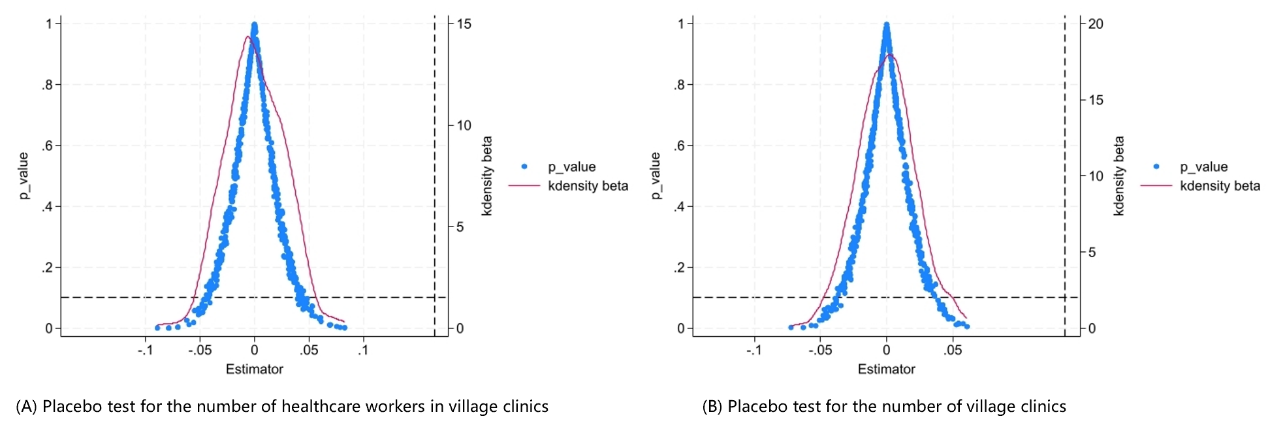


**Supplementary Figure 2.** The results of the placebo test for the impact of the CCMSC policy on healthcare service utilization in village clinics


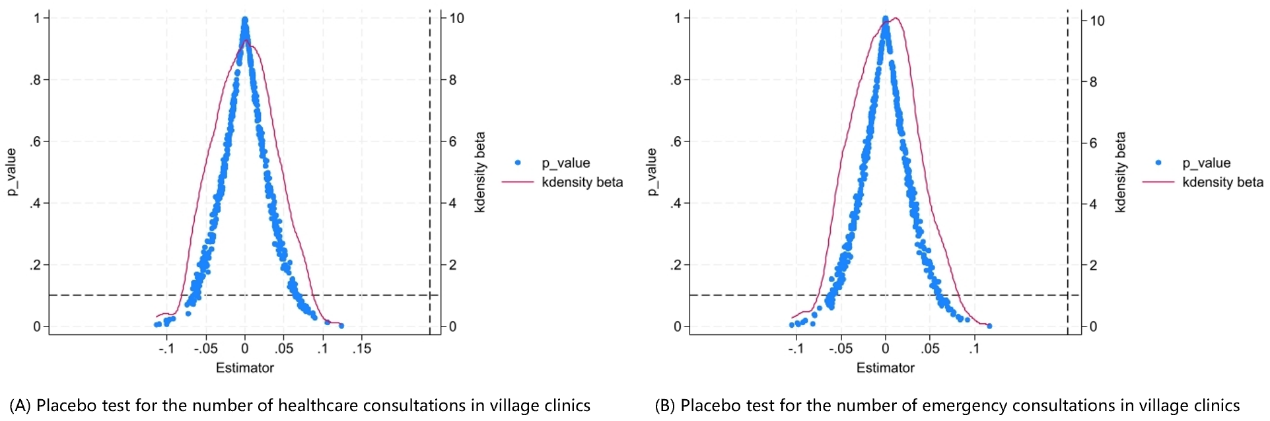

Supplement: Supplementary file 1 [file Table_1.docx]
